# Household Food Insecurity in Southeastern Iran: Severity and Related Factors

**DOI:** 10.1155/2017/7536024

**Published:** 2017-11-15

**Authors:** Zinat Mortazavi, Ahmad Reza Dorosty, Mohammad Reza Eshraghian, Mohtasham Ghaffari, Alireza Ansari-Moghaddam, Mahdi Mohammadi

**Affiliations:** ^1^Department of Community Nutrition, School of Nutritional Sciences and Dietetics, Tehran University of Medical Sciences, Tehran, Iran; ^2^Epidemiology and Biostatistics Department, School of Health, Tehran University of Medical Sciences, Tehran, Iran; ^3^Environmental and Occupational Hazards Control Research Center, School of Public Health, Shahid Beheshti University of Medical Sciences, Tehran, Iran; ^4^Health Promotion Research Center, Zahedan University of Medical Sciences, Zahedan, Iran

## Abstract

**Background:**

Today, more than one billion people globally suffer from poverty and food insecurity. This study aimed to determine the severity of and factors related to household food insecurity in Zahedan, Southeastern Iran.

**Methods:**

This cross-sectional study was conducted on a total of 2,160 households between November 2014 and December 2015. Demographic and socioeconomic data were collected through interviewing the household mothers. Household food security status was assessed through the USDA 18-item questionnaire. The data were analyzed using chi-square test, one-way ANOVA, and logistic regression model.

**Results:**

Total food insecurity in the households investigated was 58.8%. There were significant associations (*P* < 0.001) between household food insecurity status and the socioeconomic status of the households, ethnicity, education, age, and employment status of the head of the household and the mother of the household.

**Discussion:**

The results showed that more than half of the households examined suffer from food insecurity. Interventions to improve the food security status of people should be designed and implemented to improve people's knowledge, skills, and attitudes related to healthy eating and food preparation. People's access to healthy foods and knowledge of how to select healthy foods (especially on a limited budget) should also be improved.

## 1. Introduction

Food insecurity is defined as “limited or uncertain access to nutritionally adequate and safe foods or limited or uncertain ability to acquire acceptable foods in socially acceptable ways” [1–6]. Today, more than one billion people globally suffer from poverty and food insecurity [7]. Low-income households, households receiving social assistance, single-parent female-led households, renters paying rent, and children are more likely to experience food insecurity. Households with more than one child under the age of six are two times more likely to suffer from food insecurity [8]. Different studies have indicated that age, the head of household's education level, economic status, unemployment, lack of permanent employment and savings, single-parent families, ethnicity, an increase in household size, and losing food aid are all factors influencing food insecurity [9]. Food insecurity has been associated with obesity, symptoms of anxiety and depression, high-risk sexual behaviors, and negative effects during pregnancy, including low birth weight and gestational diabetes; severe obesity before pregnancy; higher weight gain in pregnancy; higher proportional weight gain; and an inability to return to prepregnancy weight; however, the evidence for the causality and direction of these associations is unclear [10, 11]. The prevalence of food insecurity in various regions of the world is varied. In a systematic review and meta-analysis conducted in Iran, the prevalence of household food insecurity was 49% [12]. In the United States in 2013, 14.3% of US households [13] and 20% of US households with children experienced food insecurity [14]. In 2014, 14% of US households were food-insecure [13]. In an urban resettlement colony of South Delhi, India, 77.2% households were food-insecure [15]. In Campina Grande, Paraíba, Brazil, 69.2% of the families were food-insecure [16]. In a study on American Indian households, 29% of children and 45% of adults were classified as food-insecure [17]. The following food insecurity rates were also observed: in northern Jordan, 32.4% [18], in Bogotá, Colombia, 76% [3], in Malaysia, 85.2% [6], in Mexican households residing near the Texas-Mexico border, 78% [19], and in Thai households, 55.8% [20]. Food insecurity occurs disproportionately among low-income households with low socioeconomic status. However, interventions and/or supplementary dietary income given to these households have not eradicated food insecurity, because insecurity is not simply due to economic poverty. Therefore, despite such interventions, food insecurity has remained an important issue in public health, even in developed countries. It has been pointed out that simply improving households' financial status or enriching children's diets is not enough to eliminate food insecurity [4]. This study aimed to determine the severity of and factors related to household food insecurity in Zahedan, Southeastern Iran.

## 2. Materials and Methods

### 2.1. Sampling Method

In this cross-sectional study, carried out from November 2014 to December 2015, 2160 Zahedanian households were investigated. The city of Zahedan was divided into five geographical areas: North, South, East, West, and Center. Two health-care centers were randomly selected from each geographical area. Each center covered a number of household blocks; these blocks were numbered. This selection was then carried out systematically according to the required sample size, with the correct number of blocks selected randomly. The households were then selected randomly from among the households covered by each block, using the household list.

### 2.2. Demographic and Socioeconomic Data

The demographic and socioeconomic data of the households were collected through interview with the mother or person responsible for preparing food for the family. These demographic characteristics included age of the head of household and his/her spouse; marital status and ethnicity of the head of household; household size; number of children; children under 18 years old. The socioeconomic characteristics included education level of the head of household and his/her spouse; occupational status of head and his/her spouse; residential house ownership status; and facilities and utilities. These were collected through a general questionnaire conducted by face-to-face interview. The facilities and utilities of life were assessed, including the possession of items including furniture, hand-woven carpets, cars, freezers, washing machines, dishwashers, microwave ovens, computer/laptops, and private houses. Based on their answers, the socioeconomic status of the households was determined. They were classified into low, moderate, and high status. Ownership of less than 3 items was considered as low, ownership of 4–6 items was considered as moderate, and possession of 7–9 items was considered as high economic status [9].

### 2.3. Household Food Insecurity

Household food insecurity was assessed through the USDA 18-item food security questionnaire [21]. The USDA household food security questionnaire which was used in this study had previously been validated in other studies conducted in Iran [22, 23]. The questionnaire, which evaluates food security status of the household over the last 12 months, was completed through interviews with the mother or person responsible for preparing food for the family. The maximum score for the questionnaire was 18. To determine the food security of the households based on the obtained score, the households were classified as 0–2: food-secure, 3–7: food-insecure without hunger, 8–12: food-insecure with moderate hunger, and 13–18: food-insecure with severe hunger [9, 23].

### 2.4. Data Analysis

Data were analyzed using SPSS 20 (SPSS Inc., Chicago, IL, USA). The prevalence of food insecurity of the households was determined and the associations of household food insecurity with socioeconomic and demographic variables were assessed. Descriptive statistical methods, one-way analysis of variance (ANOVA), and chi-square test were used. A post hoc test was used to determine the significant differences of the outcome variables among the food insecurity groups. Finally, variables related to food insecurity were entered into a multinomial logistic regression model to identify the independent risk factors of food insecurity. The significance level was considered as being <0.05.

## 3. Results

The prevalence of food insecurity in 2160 households was 58.8%, with 31.7% of households experiencing food insecurity without hunger, 19.7% food insecurity with moderate hunger, and 7.4% food insecurity with severe hunger. There were significant statistical associations between household food insecurity and ethnicity; occupation; education and age of head of the household; occupation; education and age of household's mother; household size; having children under 18; and the socioeconomic status of the household. The results obtained are shown in Table 1. As shown in Table 1, due to the limited number of other ethnic groups present in the study, groups other than Baluch were integrated into the Fars ethnicity group.

Households where the occupation of the head of household was a managerial position, physician, or university lecturer had the highest percentage of food security (95.7%) and the lowest percentage of food insecurity with moderate or severe hunger (0.0%). Households with an unemployed head had the lowest percentage of food security (9.8%) and the highest percentage of food insecurity with severe hunger (28.4%) (Table 1).

The highest percentage of food insecurity without hunger (41.2%) was observed in female head of households, where the woman was a housekeeper. In addition, there was higher food security and lower food insecurity with moderate and severe hunger in households in which the mother of the household was employed (Table 1).

The percentage of food-secure households increased with increases in the educational level of the head and the mother of household. The highest percentage of food insecurity with moderate and severe hunger was observed in households in which the head of household or mother of the household was illiterate or had only elementary education (Table 1).

Variables associated with food insecurity and food insecurity variable were entered into a multinomial logistic regression model. Variables which ultimately remained in the model were occupation of the household head; the education level of mother and the head of household; age of mother; household size; having children under 18; and socioeconomic status (Table 2).

Households in which the head was unemployed were approximately two times more likely to be food-insecure with moderate and severe hunger (OR = 1.97, 95% CI: 0.40–9.67), and the odds of food insecurity without hunger increased more than two times (OR = 2.30, 95% CI: 0.57–9.35) (Table 2).

In households with illiterate household heads, the odds of food insecurity without hunger were more than 5 times greater (OR = 5.75, 95% CI: 2.40–13.77), and the odds of food insecurity with moderate and/or severe hunger were 6 times greater (OR = 6.06, 95% CI: 2.38–15.43), compared to households in which the head of household had a university education.

The odds of food insecurity with moderate and/or severe hunger in households with an illiterate mother were more than 3 times greater (OR = 3.13, 95% CI: 1.41–6.93), compared to those whose mother had a university education (Table 2).

The food insecurity of households in the study increased with increases in household size. The household size in households with food insecurity with severe hunger (5.30 ± 1.83) was larger than in food-secure households (4.32 ± 1.65), food-insecure households without hunger, and those with moderate hunger.

The one-way analysis of variance (ANOVA) was used to investigate the association between household size and food insecurity. A statistically significant difference (*F*: 26.08, df: 3, *P* = 0.001) was observed. The Scheffe Test was used to determine the difference between groups. There was a significant statistical difference of *P* < 0.05 between household size in food-secure households and household size in food-insecure households with moderate and severe hunger and also between household size in households with food insecurity without hunger and household size in households with food insecurity with moderate and severe hunger (Table 1). The odds of food insecurity with moderate and/or severe hunger in households of 1–3 people were 52% (95% CI: 0.31–0.74), which were reduced compared to households of six or more people (Table 2).

The findings indicated that households with children under 18 experience higher food insecurity with moderate and severe hunger (20.6% and 7.7%, resp.) compared to households without children under 18 (4.4% and 0.9%, resp.) (Table 1). The odds of food insecurity with moderate and severe hunger in households with children under 18 were approximately 15 times higher (OR = 14.87, 95% CI: 4.96–44.55) than in households without children under 18 (Table 2).

Food insecurity demonstrated a positive significant association (*P* < 0.001) with the socioeconomic status of the household (Table 1).

The odds of food insecurity without hunger in households of low socioeconomic status were more than 5 times greater (OR = 5.71, 95% CI: 3.48–9.38), and the odds of food insecurity with moderate and/or severe hunger were 11 times greater (OR = 11.05, 95% CI: 5.44–22.44), compared to households of high socioeconomic status (Table 2).

Food insecurity also increased with increases in age. The mean age of the head (37.57 ± 9.95) and mother of households (32.69 ± 7.99) in households with severe food insecurity were higher than those of food-secure households and food-insecure households without hunger and with moderate hunger.

The one-way analysis of variance (ANOVA) was used to investigate the association between the age of the head of household and food insecurity. A statistically significant difference (*F*: 5.21, df: 3, *P* = 0.001) was observed. The Scheffe Test was used to determine the difference between groups. There was a significant statistical difference of *P* < 0.05 between age of the head of food-secure households and the age of the head of households with food insecurity with severe hunger and also between age of the head of households with food insecurity with severe hunger and the age of the head of households with food insecurity without hunger and with moderate hunger (Table 1).

The one-way analysis of variance (ANOVA) was used to investigate the association between the age of the mother and food insecurity. A statistically significant difference (*F*: 4.24, df: 3, *P* = 0.005) was observed. Using the Scheffe Test, the difference between groups was investigated. With *P* < 0.05, there was a significant statistical difference between the age of mothers with food insecurity with severe hunger and the age of mothers with food insecurity without hunger (Table 1).

The odds of food insecurity with moderate and/or severe hunger in mothers under 26 years of age were 60% (95% CI: 0.24–0.67), which was reduced compared to mothers over 35 years of age (Table 2).

## 4. Discussion

In the present study, food insecurity in the households investigated was 58.8%. There was a significant statistical association (*P* < 0.05) between household food insecurity and certain demographic and socioeconomic characteristics seen in households (Table 1).

In this study, 16.2% of Fars ethnic households were food-insecure with moderate hunger and 4.9% were food-insecure with severe hunger, whereas Baluch ethnic households were 27.7% and 13% food-insecure with moderate and severe hunger, respectively. The researchers did not find any study that had investigated the associations between food insecurity and ethnicity in Iran. In New Zealand, food insecurity had been associated with Maori and Oceanic ethnicities [24].

This study showed that households whose head was a university professor, physician, administrator or manager, or a military employee had the highest percentage of food security. Households with unemployed heads had the lowest percentage of food security and the highest percentage of food insecurity with severe hunger. The highest percentage of food insecurity without hunger was observed in female head of households where the woman was a housekeeper. In addition, there was higher food security and lower food insecurity with moderate and severe hunger in households in which mother of the household was employed.

Although food insecurity is not merely the result of economic poverty, it can perhaps be said that the employment of the heads of households in occupations with higher income levels and the employment of the mother of the household both increase the household income level and contribute to improving their purchasing power. Various studies in developing and developed countries have indicated that income is an important determinant of household food insecurity. In low-income families, inadequate income can lead to the inability to provide enough food for family members [6].

Studies conducted in Iran have also shown that there is a statistically significant association between the occupational status of the head of the household and food insecurity [9].

Other studies conducted on Canadian households, low-income households residing in Los Angeles, rural households in Malaysia, and women residents of California had previously shown significant associations between food insecurity and occupational status [9]. In Mexican households residing near the Texas-Mexico border, increases in the different levels of food insecurity were associated with decreases in household income and employment [19]. In an urban resettlement colony in North India, low monthly per-capita income was one of the most significant independent predictors of household food insecurity [15]. In Australia, food insecurity was associated with lower household income [25].

In this study, the percentage of food-secure households increased with increasing levels of education of the head and mother of households. The highest rate of food insecurity with moderate and severe hunger was observed in households in which the head or mother of the households was illiterate or only had elementary education.

Consistent with this study, in studies conducted in Iran [23, 26, 27], in women residing in California, households of Southern Australia, Canadian aboriginal households [9], Malaysian households [6], an urban resettlement colony in North India [15], and Jordanian households [18], there was an inversely significant association between education level and food insecurity. In Spain, occupational status, age, ethnicity, marital status, income, and education level all had significant statistical associations with food security status. Food-secure individuals had higher levels of education compared to food-insecure individuals [5].

Illiterate or low literacy restricts employment opportunities and decreases one's ability to earn money. Because of this decrease in income, the costs of providing food are also affected. Individuals' low literacy levels can also decrease their nutritional literacy level and influence all stages from the shopping basket to table (shopping, preparation, cooking, and consumption), which also can exacerbate household food insecurity [9]. Improvements in education levels lead to better employment opportunities, which can ultimately improve household food security levels [6].

In this study, consistent with other studies [6, 11, 16, 23], households with children under 18 had higher moderate and severe food insecurity, compared to households without children under 18.

In addition, with increases in household size in the households studied, food insecurity increased. The average household size in households with severe food insecurity was higher than in food-secure households. The findings of study in this field are consistent with the results of studies conducted in some of Iranian cities such as Isfahan [27], the city of Rey [9], Shiraz, and also the Asad Abadi district of Tabriz [23], which indicated significant positive associations between household size and food insecurity. In studies conducted in Indian American households [17], households of Bogotá in Colombia [3], an urban resettlement colony in North India [15], and Mexican households residing on the Texas-Mexico border [19], larger household sizes were associated with increases in food insecurity, poorer food patterns, and other consequences of food insecurity.

The composition of the household, as well as its size, affects food insecurity. With an increase in household size and a consequent increase in the number of food consumers, the minimum basic needs for foodstuff increase. In low-income households, an increase in nonfood costs can result in a decrease in spending on food, which may also be accompanied by a decrease in the food quality, food quantity, and number of meals, therefore resulting in food insecurity [15, 23].

In our study, food insecurity showed a significant positive association (*P* < 0.001) with socioeconomic status, so that 12.1% of households with low socioeconomic status had severe food insecurity, while only 1% of households with high socioeconomic status had severe food insecurity. The findings of the study are consistent with the findings of studies conducted in Iran [9, 23, 27] and other parts of the world [3, 6, 11, 24, 28, 29]. Households with higher income and better socioeconomic status have a wider food selection and can spend an appropriate portion of their income on foodstuffs [9]. In this study, consistent with certain studies conducted in Iran [9, 26] and elsewhere [5, 9], food insecurity increased with increases in age. The mean ages of the head and mother of the household in households with severe food insecurity were higher than in food-secure households. It can be said that, with an increase in age, the number of children and the household size also increase. Consequently, the quality and quantity of food assigned to each person in the household may be reduced.

## 5. Conclusion

The findings showed that more than half of the households in this study suffer from food insecurity of some level. There was higher household food insecurity in households with illiterate or low-educational level mothers or heads of the household; in households with unemployed heads; and in households with low socioeconomic status; it is thus necessary to pay more attention to these households. Short- and long-term policies should be enacted to help to improve household food security, such as helping unemployed people to find jobs; empowering women; and providing social support for low-income households.

## Figures and Tables

**Table 1:** Associations between household food insecurity and socioeconomic and demographic variables in the households studied (*n* = 2160).

Variable	Food-secure	Food-insecure without hunger	Food-insecure with moderate hunger	Food-insecure with severe hunger	Total	*χ* ^2^ test *P* value
Number (%)						
*Occupation of head of household*						
Unemployed	10 (9.8)	33 (32.4)	30 (29.4)	29 (28.4)	102 (100)	*P*<0.001
Worker	57 (16.8)	109 (32.1)	114 (33.5)	60 (17.6)	340 (100)	
Driver	53 (29.4)	60 (33.3)	46 (25.6)	21 (11.7)	180 (100)	
Professor/doctor/administrative and managerial occupations	301 (60.2)	131 (26.2)	59 (11.8)	9 (1.8)	500 (100)	
Martial	122 (60.7)	59 (29.4)	18 (9.0)	2 (1.0)	201 (100)	
Retired	45 (57.7)	26 (33.3)	5 (6.4)	2 (2.6)	78 (100)	
Self-employed	298 (40.6)	254 (34.6)	149 (20.3)	33 (4.5)	734 (100)	
Housekeeper (female heads of household)	5 (20.0)	12 (48.0)	5 (20.0)	3 (12.0)	25 (100)	
*Mother's job*						
Housekeeper	664 (36.3)	613 (33.5)	397 (21.7)	154 (8.4)	1828 (100)	*P* < 0.001
Employed	227 (68.4)	71 (21.4)	29 (8.7)	5 (1.5)	332 (100)	
*Education of household head* *Maternal education*						
Illiterate	10 (9.0)	41 (36.9)	34 (30.6)	26 (23.4)	111 (100)	*P* < 0.001
	22 (14.8)	51 (34.2)	42 (28.2)	34 (22.8)	149 (100)	
Elementary	58 (20.6)	94 (33.3)	86 (30.5)	44 (15.6)	282 (100)	
	110 (23.9)	157 (34.1)	125 (27.1)	69 (15.0)	461 (100)	
Secondary school	34 (29.5)	167 (36.8)	116 (25.6)	37 (8.1)	454 (100)	
	138 (35.7)	126 (32.6)	100 (25.8)	23 (5.9)	387 (100)	
High school & diploma	316 (40.0)	273 (34.6)	152 (19.3)	48 (6.1)	789 (100)	
	286 (42.4)	231 (34.3)	128 (19.0)	29 (4.3)	674 (100)	
University education	373 (71.2)	109 (20.8)	38 (7.3)	4 (0.8)	524 (100)	
	335 (68.5)	119 (24.3)	31 (6.3)	4 (0.8)	489 (100)	
*Having children under 18*						
Yes	832 (40.7)	635 (31)	421 (20.6)	158 (7.7)	2046 (100)	*P* < 0.001
No	59 (51.7)	49 (43.0)	5 (4.4)	1 (0.9)	114 (100)	
*Socioeconomic status*						
Low	267 (22.6)	432 (36.5)	341 (28.8)	143 (12.1)	1183 (100)	*P* < 0.001
Moderate	455 (59.1)	225 (29.2)	76 (9.9)	14 (1.8)	770 (100)	
High	169 (81.6)	27 (13.0)	9 (4.4)	2 (1.0)	207 (100)	
*Ethnicity of householder*						
Fars (and other ethnicities)	723 (48.2)	461 (30.7)	243 (16.2)	73 (4.9)	1500 (100)	*P* < 0.001
Baluch	168 (25.5)	223 (33.8)	183 (27.7)	86 (13)	660 (100)	
*Total*	891 (41.2)	684 (31.7)	426 (19.7)	159 (7.4)	2160 (100)	

Mean ± SD						
Household size	4.32 ± 1.65	4.55 ± 1.79	5.06 ± 1.83	5.30 ± 1.83	4.61 ± 1.77	*P* = 0.001
Age of household head	35.44 ± 9.45	34.35 ± 9.71	35.26 ± 9.61	37.57 ± 9.95	35.22 ± 9.61	*P* = 0.001
Maternal age	8.84 ± 31.29	8.86 ± 30.28	7.98 ± 30.61	7.99 ± 32.69	8.64 ± 30.94	*P* = 0.005

**Table 2:** Logistic regression model, socioeconomic and demographic factors affecting household food insecurity in households studied (*n* = 2160).

Variable	*n* (%)	Food insecurity without hunger^*∗*^		Food insecurity with moderate & severe hunger^*∗*^	
		*P* value	OR (95% CI)^*∗∗*^	*P* value	OR (95% CI)
*Occupation of head of household*					
Unemployed	102 (4.7)	0.241	2.30 (0.57–9.35)	0.401	1.97 (0.4–9.67)
Worker	340 (15.7)	0.809	1.16 (0.33–4)	0.855	0.87 (0.2–3.75)
Driver	180 (8.3)	0.939	0.95 (0.26–3.37)	0.385	0.51 (0.11–2.29)
Professor/doctor/administrative and managerial occupations	500 (23.1)	0.850	1.12 (0.32–3.89)	0.208	0.38 (0.08–1.70)
Martial	201 (9.3)	0.835	0.87 (0.24–3.09)	0.052	0.21 (0.04–1.01)
Retired	78 (3.6)	0.989	1.009 (0.28–3.64)	0.013	0.11 (0.02–0.63)
Self-employed	734 (34.0)	0.941	0.95 (0.28–3.22)	0.260	0.43 (0.1–1.85)
Housekeeper (women head of household)	25 (1.2)				
*Education of household head* *Maternal education*					
Illiterate	111 (5.1)	0.0001	5.75 (2.40–13.77)	0.0001	6.06 (2.38–15.43)
	149 (6.9)	0.418	1.33 (0.66–2.67)	0.005	3.13 (1.41–6.92)
Elementary	282 (13.1)	0.0001	2.68 (1.61–4.48)	0.0001	2.98 (1.63–5.43)
	461 (21.3)	0.406	1.21 (0.76–1.92)	0.002	2.57 (1.43–4.60)
Secondary school	454 (21.0)	0.0001	2.35 (1.53–3.60)	0.014	1.95 (1.14–3.34)
	387 (17.9)	0.667	0.91 (0.6–1.38)	0.024	1.88 (1.08–3.28)
High school & diploma	789 (36.5)	0.0001	1.99 (1.43–2.78)	0.003	1.98 (1.25–3.14)
	674 (31.2)	0.448	1.14 (0.81–1.59)	0.008	1.95 (1.19–3.19)
University education	524 (24.3)				
	489 (22.6)				
*Maternal age (years)*					
≤26	730 (33.8)	0.295	0.78 (0.49–1.24)	0.001	0.4 (0.24–0.67)
27–35	800 (37.0)	0.813	0.95 (0.65–1.39)	0.067	0.67 (0.44–1.02)
≥35	630 (29.2)				
*Household size (n*)					
1–3	611 (28.3)	0.154	0.76 (0.52–1.10)	0.001	0.48 (0.31–0.74)
4-5	1074 (49.7)	0.223	0.82 (0.6–1.12)	0.973	0.99 (0.71–1.38)
≥6	475 (22.0)				
*Having children under 18*					
Yes	2046 (94.7)	0.562	0.85 (0.51–1.44)	0.0001	14.87 (4.96–44.55)
No	114 (5.3)				
*Socioeconomic status*					
Low	1183 (54.8)	0.0001	5.71 (3.48–9.38)	0.0001	11.05 (5.44–22.44)
Moderate	770 (35.6)	0.0001	2.47 (1.55–3.91)	0.044	2.04 (1.01–4.09)
High	207 (9.6)				

^*∗*^Reference category is food-secure; ^*∗∗*^OR, odds ratio; CI, confidence interval.
